# Experience of Initial Symptoms of Breast Cancer and Triggers for Action in Ethiopia

**DOI:** 10.1155/2012/908547

**Published:** 2012-01-19

**Authors:** Timothy D. Dye, Solomon Bogale, Claire Hobden, Yared Tilahun, Teshome Deressa, Anne Reeler

**Affiliations:** ^1^Division of Global Health Systems and Research, Axios International 75001, Paris, France; ^2^Department of Public Health, Food Studies and Nutrition, Syracuse University, Syracuse, NY 13210-2938, USA; ^3^Radiology Department, Addis Ababa University Faculty of Medicine, Tikur Anbessa Hospital, Addis Ababa, Ethiopia; ^4^Ethiopia Breast Cancer Project, Axios Foundation, Addis Ababa, Ethiopia; ^5^Ethiopian Cancer Association, Addis Ababa, Ethiopia

## Abstract

*Objective*. This study assessed the initial experiences, symptoms, and actions of patients in Ethiopia ultimately determined to have breast cancer. *Methods*. 69 participants in a comprehensive breast cancer treatment program at the main national cancer hospital in Ethiopia were interviewed using mixed qualitative and quantitative approaches. Participants' narratives of their initial cancer experience were coded and analyzed for themes around their symptoms, time to seeking advice, triggers for action, and contextual factors. The assessment was approved by the Addis Ababa University Faculty of Medicine Institutional Review Board. *Results*. Nearly all women first noticed lumps, though few sought medical advice within the first year (average time to action: 1.5 years). Eventually, changes in their symptoms motivated most participants to seek advice. Most participants did not think the initial lump would be cancer, nor was a lump of any particular concern until symptoms changed. *Conclusion*. Given the frequency with which lumps are the first symptom noticed, raising awareness among participants that lumps should trigger medical consultation could contribute significantly to more rapid medical advice-seeking among women in Ethiopia. Primary care sites should be trained and equipped to offer evaluation of lumps so that women can be referred appropriately for assessment if needed.

## 1. Introduction

Breast cancer is an increasingly visible disease, and a rapidly growing cause of mortality, in developing countries [1]. In Africa, where breast cancer may often present at an earlier age and can progress more aggressively [2, 3], little is known about pathways and triggers for women to take action (e.g., seek medical advice) based on their recognition of symptoms.

In Ethiopia, breast cancer is typically a fatal disease with high mortality [4, 5], unlike the experience of the Western world where breast cancer is frequently treatable and with lower mortality [6]. Ethiopia has an increasingly comprehensive set of breast cancer prevention, diagnosis, and treatment interventions available for women [7] though stigma toward cancer, poor knowledge and awareness of cancer signs and treatability, and system overload continue to account for delays in reaching care [8]. An important component of the knowledge-action chain is understanding Ethiopian women's recognition of symptoms of breast cancer and their motivations for taking action. Ethiopian women typically present for care at a late stage in the disease [5], where treatment is most ineffective, and while system-related barriers to care account for a portion of that delay in access, women's attitudes and lack of awareness of breast cancer symptoms also account for a stalled initiation of action [9]. As the health system and treatments available for breast cancer in Ethiopia continually expand and are accessible to the population, more women can potentially access care at an earlier time when treatment may be more useful, provided women recognize and take action when they experience a symptom that could potentially signal breast cancer.

This evaluation includes a qualitative and quantitative assessment of the experience of participants with breast cancer who, ultimately, successfully accessed the services of the Ethiopia Breast Cancer Project (EBCP) and reports on their recognition of symptoms, their attitudes upon noticing those symptoms, and their motivations for and experiences with taking action.

## 2. Methods

This project focuses upon women who accessed care through the Ethiopia Breast Cancer Project (EBCP). EBCP aims to strengthen human resource capacity, technical competency, and advocacy and improve access to treatment for breast cancer in Ethiopia, working closely with all related departments and services of Tikur Anbessa Hospital (TAH), the Ministry of Health, and the Ethiopian Cancer Association [10]. Patients, their families, and health practitioners were interviewed as part of the larger impact assessment using semistructured interview protocols developed following open-ended ethnographic interviews and observations.

Interviewers were fluent in written and spoken Amharic and English and sequentially identified EBCP program participants to interview over a one-month span. Informed, verbal consent was obtained prior to interviewing. Interviewers participated in a one-day training session that included: study overview, ethical conduct of research, role play, and pilot test interviews and review. The teams debriefed each day with investigators for major points and discussion items for the group. Qualitative data was analyzed through theme analysis predominantly using ATLAS.ti, Version 5.5 (ATLAS.ti Scientific Software Development GmbH, Berlin, Germany), and quantitative data was analyzed using JMP, Version 8 (SAS Institute, Inc., Cary, North Carolina). Where necessary, translated English grammar in direct quotes is corrected from transcriptions to ease readability and where necessary, placed in the first person context, though content remains unchanged.

### 2.1. Sample Characteristics

In total, 55 patients directly participated in the study, in addition to 14 proxies (children, spouses, others) representing other patients, for a total of 69 patients represented. Most participants were married and female, and more than half were age 50 years or younger. About two-thirds of participants lived in Addis Ababa, and almost three-quarters of the population was Ethiopian Orthodox. Two-thirds of the participants were diagnosed with breast cancer in the immediate two years prior to the assessment. The average interview lasted 41 minutes. When compared to the clinic treatment population for the first half of 2008 from EBCP programmatic data (unpublished), the study sample did not differ significantly from the clinic treatment population on any of the following parameters: age, gender, or residence of patient.

### 2.2. Data Collection

Participants were asked in an open-ended way to tell their story of how they came to learn they had breast cancer. If necessary, participants were prompted for relative dates when they first noticed symptoms, when they first accessed care, and their navigation of the care system (see Dye et al. [11]). These narratives of participants' experiences were subsequently coded to identify symptoms, triggers for action, timing of action, and contextual factors occurring in their stories.

### 2.3. Ethical Review

This project was reviewed and approved by the Addis Ababa University Faculty of Medicine IRB. Additionally, project team members were trained in research ethics using materials from the CitiProgram (https://www.citiprogram.org/). Participant names were not collected as part of this project, and indirect identifying information was grouped and presented in general categories or in aggregate.

## 3. Results

As shown in Table 1, most participants in the assessment (82.6 percent) indicated that a lump was the first symptom (of what was to become breast cancer) that they experienced. The only other significantly mentioned symptom noted in participants' narratives of their cancer experience was a sensation of itching or burning, either on the breast or at a lymph node site. Most participants indicated that they also, ultimately, experienced subsequent symptoms, most commonly described as pain (often near or around the site of the original lump), which was experienced by 36 percent of participants. Also some participants noted that they found additional lumps (14.5 percent) subsequent to their first symptom. Nearly all participants (89.9 percent) indicated that they noticed a lump at some point prior to seeking advice for their symptoms.

Most participants did not expect that the lump they noticed was of any concern, at least initially:

*“About 2 years back, I found a small hard lump over my left breast, but since it was small and I had no pain, I was not that much concerned about it. But it kept getting bigger and bigger…(Case  47)”*

*“I came with my sister in law. She had a lump starting a year back. At first it was painless. Around 6 months later the lump become harder and painful. (Case  56)”*

*“Three years back, I started feeling sharp, tingling kind of pain in my right breast. I went to a nearby clinic and was diagnosed to have a cold, and I started oral medication. Then after some time, I developed a pea-sized hard nodule over the same breast, which was getting bigger at time passed on by. (Case  23)”*

*“I came with my sister. She told me she had a lump on her breast about two years back. We were not worried that much because it was painless. But, starting last year, the lump become painful and we took her to [a clinic]. (Case  28)”*

Similarly, as shown in Table 2, most participants (69.6 percent) ignored their symptoms, at least initially, for an average of more than one and a half years until they took some form of action. While many participants were ambiguous or uncertain for how long they ignored their symptoms, more than one-third indicated that they waited at least a year or more, some waiting as long as five or six years. This period represents the time participants waited to take action regarding their symptoms; it does not reflect typically additional delays throughout the care system once the participant made a decision to access it.

*“About two years ago, I noticed a small lump on my right breast. I just ignored it, considering it a simple swelling. After a year, I went to [Eastern Ethiopia] where my biggest child lives. I told him that I had a lump on my breast. I think he heard about breast cancer. He immediately took me to nearby clinic… (Case  37)”*

*“I saw a lump on my left breast. It was painless at that time and I thought it will resolve by its own. Finally the lump became harder… it be come [sic] very painful within two years. I went to a health center the physician examined me and told me that it is breast cancer, and I will not live more than 2 months… (Case  41)”*

Finally, participants indicated that the most common reason for initiating some form of action to address their symptoms (Table 3)—including seeking a traditional or contemporary healer—was that they experienced symptoms in addition to their primary one (42.0 percent). Further, 21.7 percent of participants indicated that they took action because of a change in their original symptom. Taken together, nearly three-fourths of all participants in the assessment were motivated to take action because of changes in or additions to their symptoms. An additional 5.8 percent indicated that family pressure motivated them to take action, and also 5.8 percent indicated that they did not deliberately take action but rather their breast cancer was detected secondarily at another health care visit.

*“Before 2 years, I started feeling sharp pain over my left breast with a little swelling. But I did not do anything about it or told anybody because I was under the consideration that it will go away by itself. But after about 4 months, I had to go to a nearby health center because the mass was getting bigger and the pain was getting worse. (Case  49)”*

*“Three years back, I started nursing my last child. After 1 month, I started to experience severe pain on my left breast. I went to a physician [who] told me that it is cold and gave me an antibiotic. Three month later I experienced a similar problem and got similar medication. After a month of this medication I noticed a small lump on the same breast. I showed it to my husband, and my sisters they told me that it simple lump and not to worry about it. After 2 and 1/2 years, I met my old friend who works in Addis Ababa as a nurse and I showed her too. She told me to go to the health center and get treated. After doing some investigation it is found to be cancer. I was shocked. (Case  27)”*

*“Two years back, I noticed a small nodule over my left breast but I was not that much concerned about it. Then when I got pregnant in that year, the mass started becoming very painful which forced to seek medical attention. Then I was told that it could be cancer and that I would get the treatment after giving birth. (Case  65)”*

## 4. Discussion

Clearly, in Ethiopia lumps are the first noticeable signs of breast cancer typically recognized by participants. Almost all of the participants in this study noticed a lump at some point and most participants also dismissed the lump, at first, as nothing to be concerned with. In fact, some participants ignored their lump for several years. In time, participants noticed more lumps or changes in symptoms (pain, itching), and that triggered them to seek advice, from a traditional healer, clinic, or other health care source. A few women were motivated to seek advice from their friends or family, and for some their cancer was discovered in the course of obtaining medical care for something else.

Several other studies have similarly found that lumps are the dominant symptom noticed by women with breast cancer and that most women find lumps as their primary symptom [12–15]. Further, breast pain was identified as a trigger for seeking care in Ghana [16], and Montazeri et al. [17] showed that painless mass was the most commonly recognized symptom in Iran, though still only 44% identified it as potentially cancer.

Studies also indicate that women in low-resource areas delay seeking care longer than women in other parts of the world, with delays of a year or more from detection of symptom to seeking advice [12, 14, 15, 17]. This delay could reflect the relatively recent (and yet incomplete, in many areas) inclusion of cancer in public health programs and awareness campaigns [18]. African women already face considerable delays in accessing care through overburdened health care systems and with limited resources; adding more than a year of delay from noticing a symptom to action increases the chances that their disease will progress significantly before care initiates.

Notably, this study included participants who, at least eventually, obtained care at the national cancer hospital in Ethiopia. Even among these women who obtained care, delay from noticing to acting on symptoms was considerable. This study is limited in that delay, symptom recognition, and the dynamics of triggers for action are unknown among women not entering the care system in Ethiopia.

Given the importance of lumps in initiating the cascade of events that could lead to breast cancer diagnosis, increasing women's knowledge of lumps as a trigger for advice may help reduce the lag time between initial notice of the symptom and eventual action in Ethiopia. Focused public campaigns and other strategies to increase awareness may be effective in promoting action when confronted with a lump (e.g., see Remennick [19]). Strengthening the capacity of the health system to respond to women's queries about lumps is also necessary [20], especially moving local primary care sites—the most common initial point of entry for breast cancer patients in Ethiopia [11]—to the basic level of capacity recommended by the Breast Health Global Initiative. Though there is debate about the effectiveness of Breast Self-Examination (BSE) in lowering breast cancer mortality (see Reeler et al. [21]), perhaps promoting breast lump awareness to encourage women to seek advice on lumps promptly, in a resource-poor country such as Ethiopia, may trigger action and entry into care sooner, while more comprehensive population-based and -accessible screening and diagnostic programs are developed and implemented over time.

## Figures and Tables

**Table 1:** First and subsequent signs of breast cancer noted among breast cancer patients, ethiopia breast cancer program, prior to taking action (*n* = 69).

*First sign noted*		

Lump	57	82.6%
Itching/burning	8	11.6%
Pain	1	1.4%

*Subsequent sign noted*		

Lumps	10	14.5%
Itching/burning	1	1.4%
Pain	25	36.2%

*Noted any lump*	62	89.9%

**Table 2:** Ignored first signs of breast cancer, patients in the Ethiopia breast cancer program (*n* = 69).

Yes	48	69.6%
No/not mentioned	21	30.4%

*Mean time ignored*	1.6 yrs	95% CI Mean: 1.0–2.2

*Ignored symptoms for how long*		
Did not ignore	21	30.4%
Under 1 yr	11	15.9%
One year	7	10.1%
Two–three years	7	10.1%
Over three years	2	2.9%
Time not specified/ambiguous	21	30.4%

**Table 3:** Triggers for action among breast cancer patients, Ethiopia breast cancer program (*n* = 69).

Change in 1st symptom	15	21.7%
Family pressure	4	5.8%
More symptoms	29	42.0%
Secondary to other care	4	5.8%
